# Professor Giampaolo Velo 31.4.1943–17.8.2017

**DOI:** 10.1007/s10787-018-0473-1

**Published:** 2018-04-13

**Authors:** K. D. Rainsford

**Affiliations:** Sheffield Hallam University, Howard Street, Sheffield, S1 1WB UK

**Figure:**
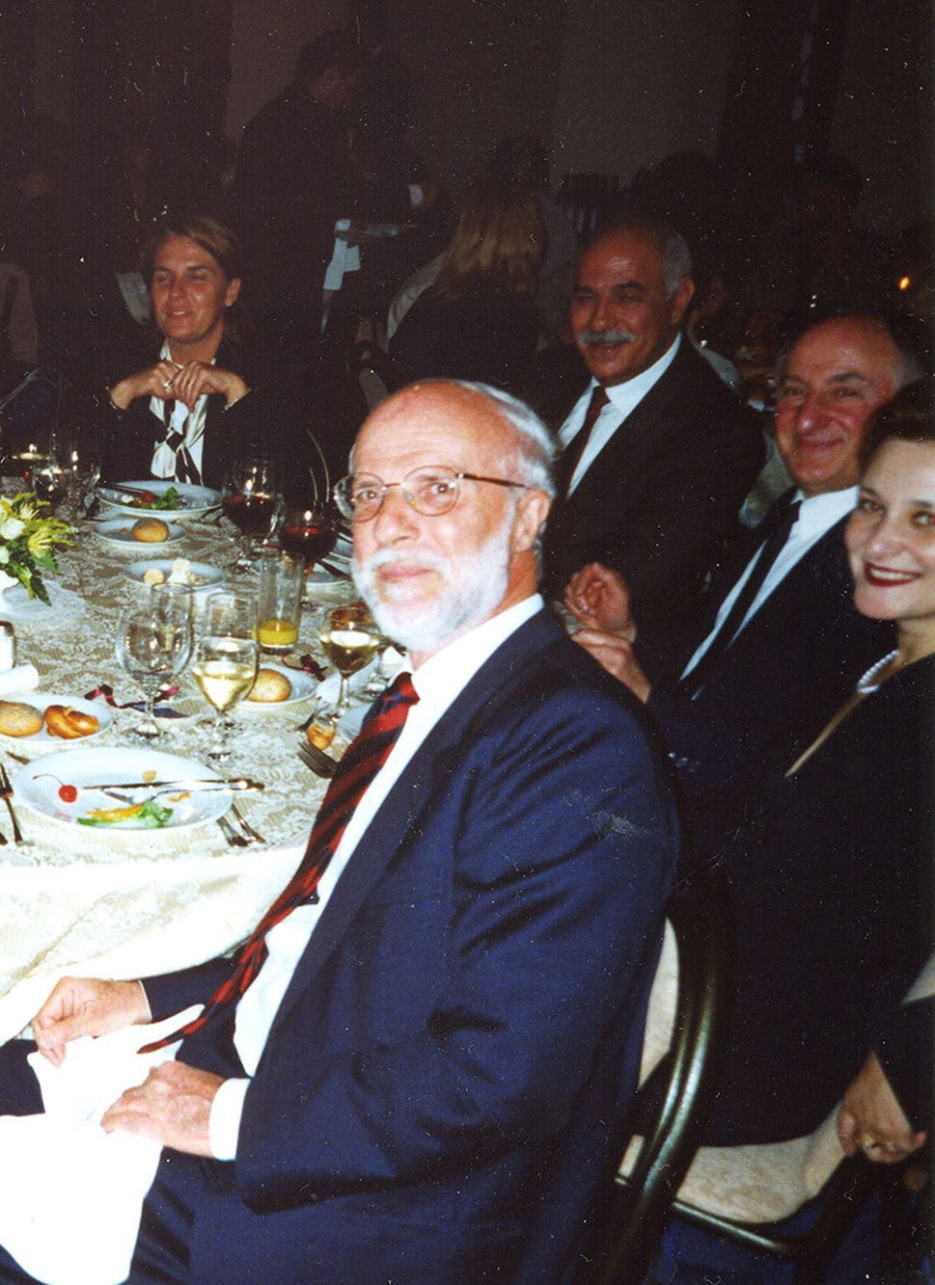
Giampaolo Velo showing geniality at a conference in Prague a decade or so ago, surrounded by friends who naturally gravitated to him

It is with much sadness that we record the untimely death of Professor Velo at the young age of 74 years. He was a foundation editor of Inflammopharmacology specializing in the clinical pharmacology and epidemiology of NSAIDs, especially in relation to adverse drug reactions, of which he was an internationally-recognized and highly regarded expert.

Giampaolo and I organized several international conferences in Verona (IT), Cambridge (UK) and Milano (IT) on “Side Effects of Anti-inflammatory Analgesic Drugs”. (Refer to “Conferences”). It was always a great pleasure to work on organizing these meetings with Giampaolo. He had a great ability to create programmes, liaise with speakers and sponsors and was a great help in organizing the venues in Italy and running the meetings as co-chairman. For this support and help in obtaining highly favourable outcomes that enjoyed wide support and recognition, as well as several successful book publications (Refer “Conference Publications”) and for his encouragement and enthusiastic support, I am immensely grateful.

**Table:** Conference proceedings

Conference details and publication of proceedings
1. Side-Effects of Anti-inflammatory/Analgesic Drugs (K.D. Rainsford and G.P. Velo, Organisers), September 1982 Publication, Book: Side-Effects of Anti-inflammatory/Analgesic Drugs. Advances in inflammation research, 6 Editors: K.D. Rainsford and G.P. Velo. Raven Press, New York, 1984
2. Side-Effects of Anti-Inflammatory Drugs (K.D. Rainsford and G.P. Velo, Organisers) Venue: University of Cambridge & Queens’ College, Cambridge (UK) 31st July to 2nd August. 1985 Publication, Book: Side-Effects of Anti-Inflammatory Drugs, 2 Part Volumes Editors: K.D. Rainsford and G.P. Velo. MTP Press, Lancaster, 1985
3. Side-Effects of Anti-Inflammatory Drugs (K.D. Rainsford and G.P. Velo, Organisers) Venue: University of Verona (Italy), 8th–11 May, 1991 Publication/Book: Side-Effects of Anti-inflammatory Drugs, 3 Editors: K.D. Rainsford and G.P. Velo. Kluwer Academic Publishers, Lancaster, 1992
4. Side-Effects of Anti-Inflammatory Drugs (K.D. Rainsford, Organiser) Venue: Sheffield Hallam University, 7th–9th August, 1995 Publication/Book: Side-Effects of Anti-Inflammatory Drugs, IV Editors: K.D. Rainsford and G.P. Velo. Kluwer Academic Publishers, Dordrecht, 1997 Publication (peer-review papers): Inflammopharmacology, Vol 3, pp 137-204 and 311-399, 1996

One of Giampaolo’s many personal attributes was his engaging and charming personality. He had an insightful human understanding, was much admired and literally “loved by all”. In these and so many ways he will be very sadly missed.

Giampaolo was born in Padova (Italy), and studied medicine and surgery at the University of Bologna from which he graduated in 1967. From 1969, he was appointed to several university posts at professorial assistant level at the Universities of Bologna-Padova (Verona). In 1986 he was appointed full Professor in Pharmacology at the University of Verona and in 1977–1999 was appointed Director of the Institute of Pharmacology of the University of Padova (Verona) and the University of Verona. It was at the beginning of this appointment that I first met Giampaolo, initially at a conference on Anti-inflammatory Drugs in Pisa organized by Professor Bertelli. Following this I frequently visited Giampaolo in his spacious institute. Entering his palatial office was a great experience—tall ceilings, grand photographs of his eminence and other great items among the grand furniture. He was supported by a remarkable secretary and had a busy team of research assistants and colleagues that gave the institute a sense of buzz and productivity. One of his valued research collaborators, was the late Dr. Roberto Milanino (Obituary, in Inflammopharmacology 2008;16:103–105), whose work on trace metal influences in inflammation has been regarded as fundamental to the understanding of the association of the effects of copper and zinc in experimentally-induced and clinically-important inflammatory conditions. Other studies on the anti-inflammatory activity of copper complexes included some novel ligands which were developed by other colleagues (chemists).

During the early 1970s Giampaolo worked at St. Bartholomew’s Hospital, London, with Professor Derek Willoughby (Obituary, in Inflammopharmacology 2005; 12:407–439). Their work involved animal studies of the effects of anti-inflammatory drugs on experimentally-induced inflammatory conditions. For this he received funding from the Wellcome Trust and NATO; later Giampaolo was to receive generous funding from NATO for organizing advanced teaching conferences on the science of inflammation and actions of anti-inflammatory drugs. These conferences were mostly held in Sicily and had wide support.

Giampaolo was a natural networker, an ability he combined with great charm. As he progressed from research on experimental pharmacology of inflammation, Giampaolo recognized the increasing concerns about the safety, or lack of it, of anti-inflammatory analgesics. For this, his involvement with agencies at the national (Veneto Region), Pan-European and WHO-Uppsala levels, led to his recognition as a leading international authority on the occurrence and developments of adverse drug reactions (ADR). Among major appointments, he was an executive member of the European Society of Pharmacovigilance (EOSP; 1998–2000), a founder member and Chairman of the European Association for Clinical Pharmacology and Therapeutics (EACPT; 1999–2003) and President of the International Society of Pharmacovigilance (ISoP; 2003–2006).

These involvements led him to be critical and characteristically conservative about ADR assessments, while managing to avoid political pitfalls especially when in an advisory role. His integrity on these aspects was widely recognized.

In more recent years, Giampaolo became interested in the problems of accumulation of pharmaceuticals and their manufacturing intermediates and metabolites in the environment, especially in surface water supplies.

Thus, in summary the contributions of Professor Giampaolo Velo to pharmacology; are that he started from the basic sciences, and achieved outstanding recognition for his work on pharma-epidemiology and drug safety monitoring. The breadth of his interests and depth of enquiries was very impressive.

He leaves his wife, Giulia, and three children, to whom he was much devoted. He was undoubtedly a very kind, hospitable and generous person to all.

**Publications—Giampaolo Velo**

Viola E, Magro L, Verlato G, Finocchio E, Leone R, Velo GP. (2016) A project on adverse drug reactions due to medication errors in hospital. Drug Safety 39(10):1025–1025 (Abstract P072).

Arzenton E, Paon V, Leone R, Apostoli P, Conforti A, Capra F, Velo GP. (2012) Acute hepatitis caused by green tea infusion: a case report. Drug Safety 35(10):944–944.

Moretti U, Lora R, Zanoni G, Tartaglia L, Ferrazin F, Velo GP, Santuccio C. (2011) Impact of non-vaccine-related reports on statistical signal detection for vaccines in spontaneous reporting databases. Drug Safety 34(10):926–926.

Leone R, Magro L, D’incau P, Minuz P, Paluanl F, Nuvolari R, Bonafini S, Velo GP. (2011) A pilot prospective observational hospital study on adverse drug reactions due to medication errors. Drug Safety 34(10):953–954.

Lindquist M, Velo GP. (2010) Erice statement 2009: communication, medicines and patient safety. Br J Clin Pharmacol 69(2):207–208.

Opri S, Leone R, Moretti U, Conforti A, D’Incau P, Magro L, Smerghetto M, Velo GP. (2010) Adverse events and adverse drug reactions in hospital observed by nurses: prospective analysis of 4608 patients. Drug Safety 33(10):922–923 (Abstract 70).

Costantini D, Leone R, Zanetti F, Grezzana M, Moretti U, Conforti A, Donati M, Velo GP. (2010) Adverse drug reactions in Italian geriatric wards. Drug Safety 33(10):923–923 (Abstract 71).

Velo GP, Minuz P. (2009) Medication errors: prescribing faults and prescription errors. Br J Clin Pharmacol 67(6):624–628.

Conforti A, Magro L, Costantini D, Opri S, Santuccio C, Venegoni M, Moretti U, Leone R, Velo GP. (2009) Haematological reactions by vaccines: data from the Italian spontaneous reporting system. Drug Safety 32(10):927–927 (Abstract 122).

Velo GP, Costantini D, Donati M, Opri S, Moretti U, Santuccio C, Venegoni M. (2009) NSAID use and safety in Italy: variation after the change of the supply status of nimesulide. Drug Safety 32(10):928–928 (Abstract 124).

Velo GP, Minuz P (2009). Medication errors: prescribing faults and prescription errors. Br J Clin Pharmacol 67(6):624–628.

Apolone G, Bassi M, Begaud B, Cascorbi I, Chiamulera C, Dodoo A, Ferro L, Ghignoni G, Isola G Juillet Y, Masotti G, Novelli G, Rizzini P, Russo P, Silano V, Tricarico G, Velo GP. (2008) Erice statement on drug innovation. Br J Clin Pharmacol (3):440–441.

Leone R, Moretti U, Conforti A, Magro L, Moschini M, Tuccori M, Cutroneo P, Motola D, Velo GP. (2008) Detecting adverse drug reactions caused by drug–drug interaction in a spontaneous reporting database. Drug Safety 31(10):938–938 (Abstract 140).

Apolone G, Bassi M, Begaud B, Cascorbi I, Ferro I, Ghignoni G, Isola G, Juillet Y, Masotti G, Russo P, Silano V, Tricarico G, Velo GP (2008) Erice statement on drug innovation. Br J Clin Pharmacol 65:440–441.

Velo GP, Motola D, Vargiu A, Magro L, Meneghelli I, Vaccheri A, Conforti A, Montanaro N. (2007) The influence of notoriety bias on ADR spontaneous reporting rate. Drug Safety 30(10):925–925.

Velo GP. (2007) Why ecopharmacovigilance? Drug Safety 30(10):932–932.

Meneghelli I, Biogi C, Iorio ML, Salvo F, Scotto S, Testi A, Velo GP. (2007) Toxicity profile of ticlopidine: unavoidable reactions? Drug Safety 30(10):952–953.

Depont F, Fourrier A, Merlière Y, Droz C, Amouretti M, Bégaud B, Bénichou J, Moride Y, Velo GP, Sturkenboom M, Blin P, Moore N; CADEUS Team (2007). Channelling of COX-2 inhibitors to patients at higher gastrointestinal risk but not at lower cardiovascular risk: the COX2 inhibitors and tNSAIDs description of users (CADEUS) study. Pharmacoepidemiol Drug Safety 16(8):891–900.

Iorio ML, Moretti U, Colcera S, Magro L, Meneghelli I, Motola D, Rivolta AL, Salvo F, Velo GP (2007). Use and safety profile of antiepileptic drugs in Italy. Eur J Clin Pharmacol. 63(4):409–415.

Leone R, Colcera S, Venegoni M, Caputi A, Conforti A, Magro L, Meneghelli I, Cocci A, Vargiu A, Velo GP. (2006) Drug-related deaths: analysis of an Italian spontaneous reporting database. Drug Saf 29(10):944–944 (Abstract 62).

Motola D, Vargiu A, Leone R, Moretti U, Scotto S, Cutroneo PM, Ros B, Velo GP, Montanaro N. (2006) Hepatic adverse drug reactions: analysis of an Italian spontaneous reporting database. Drug Saf 29(10):954–954 (Abstract 82).

Moretti U, Iorio ML, Colcera S, Passiu M, Meneghelli I, Salvo F, Motola D, De Bastiani E, Velo GP. (2006) Use and safety profile of antiepileptic drugs. Drug Safety 29(10):1003–1003 (Abstract 180).

Eland IA, Sundström A, Velo GP, Andersen M, Sturkenboom MC, Langman MJ, Stricker BH, Wiholm B; EDIP Study Group of the European Pharmacovigilance Research Group (2006). Antihypertensive medication and the risk of acute pancreatitis: the European case–control study on drug-induced acute pancreatitis (EDIP). Scan J Gastroenterol 41(12):1484–1490.

Cataldi L, Fanos V, Benini D, Riccobene F, De Mitri B, Ruggeri L, Pinna B, Sabatino G, Torcasio F, Attardo G, Tonetto P, Ziccardi MR, Zanardo V, Borgione M, Benini D, Perin M, Martano C, Velo GP. (2005) Drugs, therapeutic interventions, diseases and renal function in preterm newborn infants. A multicenter study. Pediatric Res 58(2):365–365 (Abstract 64).

Velo GP, Magro L, Cocci A, Dusi G, Ros B, Salvo F, Leone R. (2005) Statins and hepatic reactions: data from spontaneous reporting in Italy. Drug Safety 28(10):960–961 (Abstract 85).

Velo GP, Colcera S, Scotto S, Motola D, Caputi A, Conforti A. (2005) Musculoskeletal adverse drug reactions: Data from spontaneous reporting database in Italy. Drug Safety 28(10):961–962 (Abstract 87).

Velo GP, Meneghelli I, Zuliani V, Montanaro N, Venegoni M, Moretti U. (2005) Adverse drug reactions by systemic anti-bacterials: signals from spontaneous reporting in Italy. Drug Safety 28(10):961–961 (Abstract 86).

Conforti A, Magro L, Kiuru A, Velo GP, Strandell J. (2004) Ciclosporin and serious intestinal disorders. Drug Safety 27(12):924–924.

Leone R, Meneghelli I, Camerlengo T, Segat S, Moretti U, Velo GP. (2004) Domestic self-medication: an Italian attitudinal survey. Drug Safety 27(12) 943–944.

Leone R, Conforti A, Ghiotto E, Moretti U, Valvo E, Velo GP (1999). Nimesulide and renal impairment. Eur J Clin Pharmacol 55(2):151–154.

Belton KJ, Gram LF, Royer RJ, Feely J, McGettigan P, Velo GP, Conforti A, Leone R, Stricker BHC, Teixeira F, Laporte JP, Capella D, Beermann B, Lewis SC, Payne S, Rawlins MD, Wood SM. (1997) Attitude survey of adverse drug-reaction reporting by health care professionals across the European Union. Eur J Clin Pharmacol 52(6):423–427.

Franco L, Velo GP (1996). A copper-complex reduced gastric damage caused by acetylsalicylic acid and ethanol. Prostaglandins 51(5):331–338.

Pasqualicchio M, Gasperini R, Velo GP, Davies ME (1996). Effects of copper and zinc on proteoglycan metabolism in articular cartilage. Mediators Inflamm. 5(2):95–99.

Franco L, Cavallini G, Bovo P, Marcori M, Orlandi PG, Moltrer F, Vetturi B, Velo GP (1995). Gastric eicosanoid synthesis in normal subjects and alcoholics after ethanol stimulation. Ital J Gastroenterol 27(5):244–247.

Franco L, Velo GP (1995). Eicosanoid and gastroprotection by copper derivatives and NDGA. Inflamm Res 44(3):139–142.

Guglielmo L, Leone R, Moretti U, Conforti A, Velo GP (1994). Antimicrobial drug utilisation in hospitals in Italy and other European countries. Infection 22 Suppl 3:S176–S181.

Guglielmo L, Leone R, Moretti U, Conforti A, Velo GP, Vespignani S, Zappala G, Virone C, Grillo G, Barbiero A, Borsato L, Bonfanti F, Bonato D, Dona G, Barbato O, Falezza GC, Delaini C, Pedrazzoli R, Vincenzi P, Amadori F, Tomasi A, Bonifacio S, Gabrielli G, Quaglio GL, Covi MG, Stanzial M, Vettore L, Fazzini PM, Galvanini G, Sidoti G, Zennaro M, Marcon L, Faresin F, Beghelli G, Nalin G, Zavatteri G, Conti MP, Pasoli C, Pedron S, Girardello R, Cisno F, Zambotto FM, Festi G, Tommasini A, DeConti F, Cappellato G, Calvo MV, Barozzi E, Meloni GA, Tonin E, Santini GF, Martelli P, Piacentini I, Scagnelli M, Scarparo C. (1997) Aetiology and therapy of community-acquired pneumonia: a hospital study in northern Italy. Eur J Clin Pharmacol 51(6):437–443.

Franco L, Velo GP (1994) Role of nitric oxide in gastroprotection induced by copper-chelating drugs. Br J Pharmacol 111:305.

Pasqualicchio M, Davies ME, Milanino R, Velo GP (1994) Effects of copper on proteoglycan synthesis in porcine cartilage synovium co-cultures. Br J Pharmacol 111:292.

Guglielmo L, Leone R, Moretti U, Conforti A, Velo GP. (1994). Antimicrobial drug utilization in hospitals in Italy and other European Countries. Infection 22:S176–S181.

Marrella M, Gasperini R, Rizzarelli, Velo GP, Milanino R (1994). Repeated oral zinc administration in the rat—antiinflammatory effect and changes in zinc and copper status in inflamed male and female animals. In: Metals in Biology and Medicine, Vol 3. Proceedings of the Third International Symposium on Metal Ions in Biology and Medicine, 3:571–575.

Marrella M, Guerrini F, Solero PL, Tregnaghi PL, Schena F, Velo GP (1993). Blood copper and zinc changes in runners after a marathon. J Trace Elem Electrolytes Health Dis 7(4):248–250.

Franco L, Erbetti I, Velo GP (1993). Synthesis of prostaglandin E_2_ in rat liver. Pharmacol Res 28(4):367–374.

Milanino R, Frigo A, Bambara LM, Marrella M, Moretti U, Pasqualicchio M, Biasi D, Gasperini R, Mainenti L, Velo GP (1993). Copper and zinc status in rheumatoid arthritis: studies of plasma, erythrocytes, and urine, and their relationship to disease activity markers and pharmacological treatment. Clin Exp Rheumatol.11(3):271–281.

Franco L, Manara P, Erbetti I, Velo GP (1993). Anti-ulcer activity of carbenoxolone and ISF 3401 on PGE_2_ release in rat gastric mucosa. Pharmacol Res;27(2):141–150.

Cristofori P, Terron A, Marella M, Moretti U, Pasqualicchio M, Velo GP, Milanino R (1992). Copper supplementation in the rat: preliminary observations on the clinical, hematological and histopathological profile. Agents Actions. Spec No:C118–C120.

Franco L, Velo GP. (1992) Effect of copper on gastric eicosanoid production. Pharmacol Res 25:240–241.

Milanino R, Marerella M, Moretti U, Pasqulicchio M, Gasperini R, Velo GP. (1992) Antiinflammatory activity of indomethacin in copper deficient rats. Pharmacol Res 25:254–255.

Milanino R, Deganello A, Marrella M, Michielutti F, Moretto U, Pasqualicchio M, Tamassia G, Tato L, Velo GP. (1992). Oral zinc as initial therapy in Wilson’s-Disease—2 years of continuous treatment in a 10-year-old child. Acta Paediatrica 81(2):163–166.

Marrella M, Gasperini R, Milanino R, Moretti U, Pasqualicchio M, Gandini G, Travesini E, Vaccari R, Velo GP (1992). Influence of sex and age on blood copper and zinc. Metal Ions Biol Med 2:426–427.

Fracasso ME, Franco L, Gasperini R, Velo GP (1992). NSAIDs on liver microsomal monooxygenase system and products of oxidative metabolism of arachidonic acid. In: Side-effects of anti-inflammatory drugs. 3:204–210.

Franco L, Erbetti I, Bacchini P, Velo GP (1992). The role of copper in preventing gastric damage by acetylsalicylic acid. In: Side effects of anti-inflammatory drugs, 3:359–362.

Milanino R, Frigo A, Marrella M, Bambara LM, Moretti U, Biasi D, Pasqualicchio M, Mainenti I, Velo GP (1992). Effect of NSAID therapy on plasma, whole blood cell (BC), and 24 h urine zinc in patients with rheumatoid arthritis (RA). In: Side-effects of anti-inflammatory drugs 3:363–366.

Cavallini G, Brocco G, Bovo P, Frulloni L, DiFranceso V, Franco L, Velo GP, Psilogenis M, Nazzari M (1991). Effects of sulglytode on PGE_2_ and gastric mucus in healthy volunteers. Acta Therapeutica 17(2):173–178.

Velo GP (1991) Pharmacologic rationale for the use of non-steroidal anti-inflammatory agents in acute pain. Minerva Anestesiol. 57(11):1275–1276.

Conforti A, Caliceti P, Sartore L, Schiavon O, Veronese F, Velo GP (1991). Anti-inflammatory activity of monomethoxypolyethylene glycol superoxide dismutase on adjuvant arthritis in rats. Pharmacol Res 23(1):51–56.

Fracasso ME, Leone R, Cuzzolin L, Del Soldato P, Velo GP, Benoni G (1990). Indomethacin induced hepatic alterations in mono-oxygenase system and faecal Clostridium perfringens enterotoxin in the rat. Agents Actions 31(3–4):313–316.

Gasperini R, Leone R, Velo GP, Fracasso ME (1990). The inhibition of hepatic microsomal drug metabolism in rats by non-steroidal anti-inflammatory drugs. Pharmacol Res 22 Suppl 3:115–116.

Velo GP, Milanino R (1990). Nongastrointestinal adverse reactions to NSAID. J Rheumatol Suppl. 20:42–45.

Cuzzolin L, Caliceti P, Conforti A, Veronese FM, Velo GP, Benoni G (1990). Are bacterial toxins involved in arthritis induced in rats? Acta Physiol Hung 75 Suppl:71–72.

Cuzzolin L, Caliceti P, Conforti A, Veronese FM, Benoni G, Velo GP (1990). Intestinal bacterial flora and lesions in arthritic rats: effect of antiinflammatory drug. Acta Physiol Hung 75 Suppl:69–70.

Pasqualicchio M, Marrella M, Moretti U, Velo GP, Deganello A, Tomelleri G, Milanino R (1990). Oral zinc sulphate treatment in Wilson’s disease. Acta Physiol Hung 75 Suppl:233–234.

Franco L, Cavallini G, Brocco G, Orlandi PG, Manara P, Velo GP (1990). 40% but not 20% ethanol is an irritant able to evoke gastric release of PGE_2_ in man. Acta Physiol Hung 75 Suppl:113–114.

Conforti A, Leone R, Moretti U, Sartori M, Velo GP (1990). Multicenter study of hospital adverse drug reactions. Acta Physiol Hung 75 Suppl:63–64.

Marrella M, Moretti U, Pasqualicchio M, Velo GP, Frigo A, Trevisani E, Bambara LM, Milanino R (1990). Plasma and total blood cell copper in rheumatoid arthritis. Agents Actions 29(1–2):120–121.

Fracasso ME, Leone R, Cuzzolin L, Del Soldata P, Velo GP, Benoni G. (1990) Indomethacin induced hepatic alterations in mono-oxygenase system and fecal Clostridium-perfringens enterotoxin in the rat. Agents and Actions 31(3–4):313–316.

Marella M, Milanino R, Morelli U, Deganello A, Velo GP (1989) One year of oral zinc-sulphate therapy in a child with Wilson’s disease. Pharmacological Research 21(4):489–490.

Velo GP, Cavallini G, Brocco G, Orlandi PG, Franco L. (1989) Gastric cytoprotection. Prostanoids and Drugs 177:153–160.

Pasqualicchio M, Milanino R, Marrella M, Moretti U, Tomelleri G, Velo GP (1989). Two and half years of oral zinc sulphate therapy in an adult patient with Wilson’s disease. Pharmacol Res 21 Suppl 1:151–152.

Milanino R, Marrella M, Moretti U, Velo GP, Deganello A, Ribezzo G, Tatò L. (1989) Oral zinc sulphate as primary therapeutic intervention in a child with Wilson disease. Eur J Pediatr 148(7):654–655.

Moretti U, Cavallini G, Milanino R, Brocco G, Concari E, Riela A, Marrella M, Bertelli G, Pelle C, Scuro LA, Velo GP. (1988) Concerning a possible relationship between chronic pancreatitis and hypocupremia. Ital J Gastroenterol 20 (2):107–107.

Boner AL, Velo GP, Piacentini GL, Peroni DG (1988) Theophylline-induced inhibition of pulmonary defense. Annals Allergy 60(2):183–183.

Milanino R, Moretti U, Concari E, Marrella M, Velo GP (1988). Copper and zinc status in adjuvant-arthritic rat: studies on blood, liver, kidneys, spleen and inflamed paws. Agents Actions 24(3–4):365–376.

Milanino R, Marrella M, Moretti U, Concari E, Velo GP (1988). Copper and zinc status in rats with acute inflammation: focus on the inflamed area. Agents Actions. 24(3–4):356–364.

Marrella M, Moretti U, Concari E, Velo GP, Milanino R. (1988) Changes of Cu & Zn in carrageenan pleurisy and paw-oedema in rat. Pharmacol Res Commun 20(7):629–630.

Cuzzolin L, Tomelleri G, Bongiovanni LG, Benoni G, Velo GP. (1988) Phenytoin-phenobarbital interaction: importance of free plasma phenytoin monitoring. Pharmacol Res Commun 20(7):627–628.

Fracasso, Cuzzolin L, DelSoldato P, Leone R, Velo GP, Benoni G. (1987) Multisystem toxicity of indomethacin—effects on kidney, liver and intestine in the rat. Agents Actions 22(3–4):310–313.

Moretti U, Cavallini G, Milanino R, Brocco G, Concari E, Riela A, Marrella M, Bertelli G, Pelle C, Togni M, Scuro LA, Angelini G, Velo GP. (1987) Concerning a possible relationship between chronic pancreatitis and hypocupraemia. Dig Dis Sci 32(10):1177.

Graziani G, Abbiati GA, Dolfini E, Testa R, Velo GP. (1987) Pharmacokinetic, pharmacodynamic, and toxicological properties of naproxen gel in laboratory animals. Current Therap Res Clin and Exper 42(3):480–490.

Fracasso ME, Cuzzolin L, Del Soldato P, Leone R, Velo GP, Benoni G. (1987) Multisystem toxicity of indomethacin: effects on kidney, liver and intestine in the rat. Agents Actions 22(3–4):310–313.

Minuz P, Lechi A, Arosio E, Degan M, Capuzzo MG, Lechi C, Corsato M, Dalla Riva A, Velo GP. (1987) Antihypertensive activity of enalapril. Effect of ibuprofen and different salt intakes. J Clin Hypertens 3(4):645–653.

Conforti A, Franco L, Milanino R, Velo GP, Bocc + 53u E, Largajolli E, Schiavon O, Veronese FM. (1987)PEG superoxide dismutase derivatives: anti-inflammatory activity in carrageenan pleurisy in rats. Pharmacol Res Commun 19(4):287–294.

Velo GP, Minuz P, Riela A, Brocco G, Degan M, Franco L, Cavallini G. (1987) Gastric and systemic effects of carbenoxolone and its derivative ISF 2715 in humans. Adv Prostaglandin, Thromboxane, Leukotriene Res. 17A:361–365.

Milanino R, Cassini A, Conforti A, Franco L, Marrella M, Moretti U, Velo GP. (1986) Copper and zinc status during acute inflammation: studies on blood, liver and kidneys metal levels in normal and inflamed rats. Agents Actions 19(3–4):215–223.

Minuz P, Cavallini G, Brocco G, Degan M, Jeunet F, Kunovits G, Riela A, Velo GP. (1986) Effect of carprofen and indomethacin on gastric function and the content of prostaglandins E_2_ and F_2 alpha_ in human gastric juice. Hepato-gastroenterology 33(1):20–22.

Benoni G, Arosio E, Raimondi MG, Pancera P, Lechi A, Velo GP. (1985) Pharmacokinetics of ceftazidime and ceftriaxone and their penetration into the ascitic fluid. J Antimicrob Chemother. 16(2):267–273.

Minuz P, Degan M, Covi G, Lechi C, Velo GP, Lechi A. (1985) Urinary 6-keto-PGF_1 alpha_ after captopril and indomethacin: possible contribution of PGI_2_ to the antihypertensive mechanism of ACE inhibitors. Adv Prostaglandin Thromboxane Leukotriene Res. 13:199–202.

Milanino R, Cassini A, Franco L, Conforti A, Marrella M, Velo GP. (1985) Copper metabolism in the acute inflammatory process and its possible significance for a novel approach to the therapy of inflammation. Int J Tissue React. 7(6):469–474.

Minuz P, Cavallini G, Angelini GP, Lechi A, Brocco G, Riela A, Scuro LA, Velo GP.(1984) Carbenoxolone and prostaglandin E2 and F2 alpha gastric juice levels in man. Pharmacol Res Commun. 16(9):875–884.

Benoni G, Arosio E, Raimondi MG, Apolloni E, Passarella E, Lechi A, Velo GP. (1984) Distribution of ceftazidime in ascitic fluid. Antimicrob Agents Chemother. 25(6):760–763.

Benoni G, Cuzzolin L, Puchetti V, Velo GP. (1984) Penetration of ceftazidime in human pericardial fluid and lung tissue. Pharmacol Res Commun. 16(3):295–301.

Conforti A, Franco L, Milanino R, Totorizzo A, Velo GP. (1983) Copper metabolism during acute inflammation—studies on liver and serum copper concentrations in normal and inflamed rats. Br J Pharmacol 79(1):45–52.

Velo GP, Franco I, Conforti A, Milanino R. (1983) Copper and its role in the modulation of the inflammatory process as a new approach to the anti-arthritic therapy. Scan J Rheumatol, 26, (abstract).

Veronese FM, Boccù E, Schiavon O, Velo GP, Conforti A, Franco L, Milanino R. (1983) Anti-inflammatory and pharmacokinetic properties of superoxide dismutase derivatized with polyethylene glycol via active esters. J Pharm Pharmacol. 35(11):757–758.

Conforti A, Franco L, Menegale G, Milanino R, Piemonte G, Velo GP. (1983) Serum copper and ceruloplasmin levels in rheumatoid arthritis and degenerative joint disease and their pharmacological implications. Pharmacol Res Commun. 15(9):859–867.

Conforti A, Franco L, Milanino R, Totorizzo A, Velo GP. (1983) Copper metabolism during acute inflammation: studies on liver and serum copper concentrations in normal and inflamed rats. Br J Pharmacol. 79(1):45–52.

Conforti A, Franco L, Milanino R, Velo GP. (1982) Copper and ceruloplasmin (Cp) concentrations during the acute inflammatory process in the rat. Agents Actions. 12(3):303–307.

Boccù E, Velo GP, Veronese FM. (1982) Pharmacokinetic properties of polyethylene glycol derivatized superoxide dismutase. Pharmacol Res Commun. 14(2):113–120.

Conforti A, Franco L, Milanino R, Velo GP. (1981) Serum copper concentration and ceruloplasmin activity during carrageenan foot edema in rat. Br J Pharmacol 72(1):P137–P138.

Milanino R, Velo GP. (1981) Multiple actions of copper in control of inflammation: studies in copper-deficient rats. Agents Actions Suppl. 8:209–230.

Benoni G, Franco L, Conforti A, Totorizzo A, Velo GP. (1981) Pharmacokinetics of cefuroxime and cefoxitin in experimentally induced pleurisy in the rat. G Ital Chemioter. 28(1–2):33–39.

Milanino R, Conforti A, Fracasso ME, Franco L, Leone R, Passarella E, Tarter G, Velo GP. (1980) The acute inflammatory process in copper-deprived rats. Agents Actions Suppl. 7:224–232.

Berti T, Bozzini L, Martini N, Minelli Bertazzoni E, Scanagatta A, Velo GP. (1979) Therapeutic hospital formulary for a better use of drugs. Pharmacol Res Commun 11(5):379–387.

Milanino R, Conforti A, Fracasso ME, Franco L, Leone R, Passarella E, Tarter G, Velo GP (1979). Concerning the role of endogenous copper in the acute inflammatory process. Agents Actions 9(5–6):581–588.

Velo GP, Fracasso ME, Leone R. Prostaglandins and inflammation. G Ital Chemioter. 1979;26(1–2):85–90.

Velo GP. (1978) Prostaglandins and inflammation. Agents Actions 8(1–2):158–158.

Milanino R, Mazzolini S, Passarella E, Tarter G, Velo GP. (1978) Carrageenan edema in copper-deficient rats. Agents Actions 8(6):618–622.

Milanino R, Passarella E, Velo GP. (1978) Adjuvant arthritis in young copper-deficient rats. Agents Actions 8(6):623–628.

Milanino R, Mazzoli S, Passarella E, Tarter G, Velo GP (1978). Carrageenan oedema in copper-deficient rats. Agents Actions. 8(6):618–622.

Milanino R, Passarella E, Velo GP. (1978) Adjuvant arthritis in young copper-deficient rats. Agents Actions. 8(6):623–628.

Abdulla SE, Martelli EA, Bramm E, Franco L, Velo GP. (1977) Effect of calcitonin on different inflammatory modes. Agents Actions 7(5–6):533–538.

Velo GP, Dedastini G, Nogarin L, Abdulla SE. (1976) Anti-inflammatory effect of calcitonin. Agents Actions 6(1–3):284–284.

Giroud JP, Willoughby DA, Velo GP. (1976) Future trends in inflammation. 2. Proceedings of an international meeting on inflammation of inflammation, Paris, 21–24, May 1975—general closing remarks. Agents Actions 6(1–3):377–380.

Abdulla SE, De Bastiani G, Nogarin L, Velo GP.(1975) Effect of calcitonin on carrageenan foot oedema. Agents Actions. 5(4):371–373.

Willoughby DA, Giroud JP, Velo GP. (1974) Progress in inflammation applicable to chronic evolutive polyarthritis. Brux Med. 54(3):135–138.

Giroud JP, DiRosa M, Velo GP, Timsit J, Willoughby DA. (1974) Pharmacological analysis of cellulose sulfate on lymphatic system. J Pharmacologie 5(1):128–128.

Giroud JP, DiRosa M, Timsit J, Velo GP, Willoughby DA. (1974) Pharmacological study of effects of cellulose sulfate on lymphatic system of rat. J Pharmacologie 5(3):309–320.

Velo GP, Dunn CJ, Giroud JP, Timsit J, Willoughby DA. (1973) Distribution of prostaglandins in inflammatory exudate. J Pathol. 111(3):149–158.

Velo GP, Spector WG. (1973) The origin and turnover of alveolar macrophages in experimental pneumonia. J Pathol. 109(1):7–19.

Velo GP, Dunn CJ, Giroud JP, Timsit J, Willoughby DA (1973) Distribution of prostaglandins in inflammatory exudate. J Pathol 11(3):149.

Velo GP, Spector WG. (1973) Origin and turnover of alveolar macrophages in experimental pneumonia. J Pathol 109(1):7.

Velo GP, Bertoni F, Capelli A, Martinelli G. (1972) Lysosomes as mediators of parenchymal lesions in adjuvant-induced arthritis in rats. J Pathol. 106(3):201–205.

Velo GP, Bertoni F, Capelli A, Martinelli G. (1971) Lysosomes as mediators of parenchymal lesions in adjuvant-induced arthritis in rats. J Pathol. 104(3):3–4.

Velo GP, Capelli A, Matinelli G, Bertoni F. (1971) Lysosomes as mediators of parenchymal lesions in adjuvant-induced arthritis in rats. Naunyn-Schmiedebergs Arch Pharmakol 269(2–4):484.

Velo GP, Capelli A, Martinelli G, Bertoni F. (1970) Articular histochemical findings in arthritis induced by Freund’s adjuvant in rats. Comparison with human rheumatoid arthritis. Boll Soc Ital Biol Sper. 30;46(8):395–397.

Bertoni F, Capelli A, Velo GP. (1969) Morphological and histochemical studies of articular lesions induced in rats with Freund’s adjuvant. II. Findings in the paws at the level of the articular cartilage and synovial membrane. Fracastoro. 62(6):589–594.

Velo GP, Capelli A, Bertoni F. (1969) Morphogical and hiochemical studies of articular lesions induced in rats with Freund’s adjuvant. I. Findings at the level of the caudal vertebral articulations]. Fracastoro 62(6):582–588.

Bertoni F, Velo GP (1969). Finding of cardiac lesions in rats with “adjuvant arthritis”. Boll Soc Ital Biol Sper 45(1):41–44.

Velo GP (1969). Preliminary research on the activity of anti-inflammatory drugs in arthritis induced by adjuvant in rats. Boll Soc Ital Biol Sper. 45(1):39–41.

Velo GP (1969). Erythrosedimentation as a parameter of arthritis induced with adjuvant in rats. Boll Soc Ital Biol Sper. 5;45(1):36–39.

Bertazzoni E, Velo GP, Vettori G, Berti T (1968). Antibacterial activity of rifampicin on faecal flora in rats. Antibiotica. 6(3):125–33.

